# Predicting Pediatric Urological Surgery Duration Through Multimodal Patient-Physician Feature Fusion: Deep Learning Framework Incorporating Clinical Text Embedding

**DOI:** 10.2196/82329

**Published:** 2026-04-28

**Authors:** Yonggen Zhao, Ruoge Lin, Yiying Sun, Lingdong Chen, Jian Huang, Guangjie Chen, Zhu Zhu, Gang Yu

**Affiliations:** 1 National Clinical Research Center for Children and Adolescents' Health and Diseases, Children's Hospital, Zhejiang University School of Medicine, 3333 Binsheng Rd, Hangzhou, 310052, China, 86 13588773370; 2 Sino-Finland Joint AI Laboratory for Child Health of Zhejiang Province, Hangzhou, China; 3 College of Biomedical Engineering & Instrument Science, Zhejiang University, Hangzhou, China; 4 Department of Urology, Children's Hospital, Zhejiang University School of Medicine, Hangzhou, China

**Keywords:** surgical duration prediction, operating room management, deep neural network, multihead perceptron, medical informatics

## Abstract

**Background:**

Accurate prediction of surgical duration is critical for optimizing operating room scheduling and resource allocation. Existing models, however, exhibit limited applicability in pediatric urology due to the unique anatomical and developmental characteristics of children.

**Objective:**

This study aimed to develop and validate a specialty-tailored prediction framework for estimating the duration of pediatric urological surgeries.

**Methods:**

We integrated multisource heterogeneous data, encompassing patient demographics, surgical details, surgeon-specific features, and electronic medical record narratives, to develop a customized prediction system. Large language model techniques were used to extract semantic representations from unstructured clinical text, while a multihead perceptron architecture enabled the efficient fusion of structured and unstructured features. Pediatric-specific clinical variables, such as developmental stage and the severity of urinary tract malformations, were explicitly modeled to capture their impact on surgical duration.

**Results:**

The proposed approach achieved a mean absolute error of 11.39 minutes and a root mean square error of 15.58 minutes, markedly outperforming existing methods. Comparative analyses demonstrated that the Qwen-based structured preprocessing with text embeddings provided superior feature representation, surpassing both traditional long short-term memory and direct Embedding-3 approaches. Feature importance analysis identified the primary surgical procedure, surgical plan, and preoperative diagnosis as dominant predictive factors.

**Conclusions:**

By combining innovative feature engineering with a tailored model architecture, the proposed framework substantially improves the accuracy of surgical duration prediction in pediatric urology. These findings offer robust technical support for precision operating room scheduling and hold significant clinical value in enhancing the efficiency of surgical resource utilization.

## Introduction

### Background and Motivation

As one of the hospital’s most resource-intensive departments, the operating room plays a pivotal role in determining the institution’s financial health and overall success [1,2]. Optimizing operating room scheduling under constraints of available bed capacity and human resources has become a key lever for improving health care service efficiency. Within this process, accurate prediction of surgical duration is a prerequisite for effective scheduling decisions [3-6]. However, due to the presence of multiple sources of uncertainty, such as patient heterogeneity (eg, disease complexity and anatomical variation), intraoperative contingencies (instrument failure and unexpected bleeding), and dynamic adjustments to the surgical plan [7,8], predicting surgical duration remains a major challenge in perioperative management [9,10]. Traditional estimation methods largely rely on the surgeon’s personal experience or simple historical averages [11,12]. These approaches lack standardized classification of surgical procedures and fail to systematically integrate dynamic variables, such as patient-specific characteristics, team collaboration efficiency, and equipment turnover rates, leading to substantial discrepancies between estimated and actual durations [13,14]. Such inaccuracies can trigger a cascade of operational disruptions. The underestimation of duration can lead to staff fatigue, congestion in emergency channels [15], and prolonged patient waiting and fasting times, thereby increasing the risk of adverse events. Overestimation, on the other hand, results in idle resource time. More critically, errors in estimating a single case can be amplified through the scheduling system, ultimately causing mismatches in staffing, cost, and heightened dissatisfaction among patients and their families [16,17]. Therefore, developing more accurate and reliable methods for surgical duration prediction is both important and urgent.

In recent years, machine learning has made significant advances in surgical duration prediction [18,19]. This research can be broadly categorized by the types of features used.

### Patient- and Procedure-Oriented Studies

These methods mainly rely on patient characteristics and surgical information, leveraging demographic data, preoperative physiological indicators, and procedural details for predictive modeling. Commonly used algorithms include multilayer perceptrons (MLPs) and Extreme Gradient Boosting, with some studies incorporating real-time intraoperative parameters, such as heart rate and bispectral index, to enable dynamic predictions [20-23]. Key predictive factors identified in these studies include patient BMI, surgical type, and procedural complexity, with certain models achieving prediction errors—the mean absolute error (MAE) within a clinically acceptable margin of 30 minutes.

### Surgeon- and Procedure-Oriented Studies

Such studies emphasize surgeon-related and procedural factors, particularly the impact of operator experience, surgical techniques, and case urgency on operative duration. Ensemble methods, such as bagged decision trees, gradient boosting trees, and Extreme Gradient Boosting, have demonstrated strong performance in this domain, especially for single-specialty or emergency procedures, with reported MAEs as low as 14.9 minutes [24-28]. Feature importance analyses consistently highlight surgical complexity and surgeon proficiency as dominant predictors.

### Comprehensive Studies Integrating Patient, Doctor, and Surgical Information

The most comprehensive approaches integrate multimodal clinical data, combining patient-specific variables, surgeon expertise, and procedural factors, to optimize predictive accuracy. Random forests and MLPs are frequently adopted in such frameworks, while some studies have further improved performance through specialized department-specific modeling or 2-stage prediction architectures, achieving MAEs below 16 minutes in targeted clinical settings [29-31]. Emerging techniques, including computer vision–based analysis of surgical videos and large language models (LLMs) (eg, GPT-4), are also gaining traction, demonstrating promising capabilities in processing unstructured clinical narratives for duration estimation [32,33].

Despite the diversity of approaches in existing studies, several critical limitations persist. A primary issue lies in feature engineering, where current methods predominantly rely on structured data fields, such as procedure names and diagnostic codes, while largely neglecting the wealth of information contained in unstructured clinical notes. These free-text entries, which include detailed condition descriptions and laboratory results, often capture patient-specific variations that could significantly enhance prediction accuracy. Furthermore, existing models demonstrate limited applicability to pediatric surgical contexts. Adult-derived models fail to account for pediatric-specific physiological considerations, including developmental variations in organ systems and anatomical structures. This oversight is particularly problematic, given the narrower intraoperative safety margins in pediatric patients due to their reduced physiological reserves [12], which demands greater predictive precision.

This study focuses on pediatric urological surgery and develops a novel specialty-oriented prediction method. Compared with adult procedures, pediatric urology presents unique clinical challenges: the patient age range spans from newborns to adolescents, with marked developmental variations in physiological parameters, and differences in urinary tract development and malformation severity influence the level of operative precision required. These factors make adult-trained models poorly transferable to pediatric cases.

To address these issues, we designed an end-to-end prediction system, which integrates multimodal clinical data, including patient characteristics, surgical information, surgeon attributes, and clinical narratives, to model and accurately predict pediatric urological surgery duration. We innovatively used LLM techniques to automatically process electronic medical record (EMR) text containing detailed case descriptions, examination findings, and physiological features, extracting and embedding unstructured clinical information efficiently. In addition, we proposed a multihead MLP architecture for heterogeneous data fusion, allowing categorical, numerical, and text feature vectors to be processed independently before integration, outperforming traditional single-head MLP approaches. We further applied permutation importance analysis to identify the most influential predictors, including the lead surgeon, primary surgical procedure, preoperative diagnosis, and pediatric-specific disease characteristics (eg, prepuce characteristics and testicular characteristics). Comparative and ablation experiments demonstrated that our method substantially improved prediction accuracy, achieving an MAE of 11.39 minutes and a root mean square error (RMSE) of 15.58 minutes—significantly better than the existing work. Our model exhibits clear advantages in predicting pediatric urological surgery duration, providing reliable support for precise operating room scheduling.

## Methods

### Data Acquisition and Preprocessing Flow

The data used in this study were obtained from surgical cases performed at the Urology Surgery Center of the Children’s Hospital, Zhejiang University School of Medicine, between May 22, 2024, and July 10, 2025. The hospital is an independent tertiary pediatric medical center. All surgical information was extracted from the hospital information system, yielding a total of 4526 cases. These encompassed 4 major categories of pediatric urological procedures: penile lengthening (n=2067, 45.7%), orchidopexy (n=937, 20.7%), laparoscopic high ligation of the processus vaginalis (n=1158, 25.6%), and circumcision (n=364, 8%).

### Feature Extraction

In this study, the duration of surgery was defined as the time interval commencing from “anesthesia induction” and concluding when the patient left the operating room. This definition encompasses not only the surgical procedure itself but also key stages, such as anesthesia induction, preoperative preparation, and postoperative transfer prior to the patient’s departure from the operating room. As a result, it more comprehensively reflects the actual occupancy period of the operating room. When compared with simplified definitions, such as “from skin incision to suture completion” or “from entry to exit of the operating room,” the time frame used in this study more accurately reflects the real-world workflow under multidisciplinary collaboration and holds direct guiding significance for hospital surgical scheduling and resource allocation [34]. For example, precise prediction of this period facilitates the rational arrangement of consecutive surgeries, optimizes the coordination between the anesthesia team and the recovery room, and mitigates idle time or congestion arising from estimation errors. All duration data were obtained from the hospital information system, guaranteeing the consistency and traceability of the definition. Considering that previous research has suggested that the duration of surgery generally follows a log-normal distribution [35,36], we standardized the target variable (with a mean of 0 and an SD of 1) to improve the stability of model training and the balance of feature scales.

Additional predictor variables extracted from the information system included patient age, patient weight, primary surgical procedure, lead surgeon features, surgical plan, preoperative diagnosis, and case-specific characteristics, including preoperative preparation details. All extracted variables were used by the prediction model.

Patient age and weight are continuous variables. Patient age was recorded in the format “x years x months” and was converted to years, followed by standardization. If the age was missing, it was replaced with the age mentioned in the “case characteristics and preoperative preparation” notes. Patient weight, recorded in kilograms, was also standardized. If missing or abnormal, it was imputed using the average weight of patients in the same age group.

Primary surgical procedure and lead surgeon features are categorical variables. The primary surgical procedure is a single-category variable referring to the main pediatric urological surgery performed for the patient, with only 1 such designation per case. This study primarily covered 4 main procedure types: penile lengthening, orchidopexy, laparoscopic high ligation of the processus vaginalis, and circumcision. This variable was one-hot encoded. The lead surgeon data were expanded into 3 distinct features to capture specific physician characteristics: name, years of practice, and annual surgical volume. The surgeon name is a single-category variable representing the primary attending specialist and was processed using one-hot encoding. Years of practice is a continuous numeric variable representing the duration of the surgeon’s professional experience. Annual surgical volume is a discrete numeric variable indicating the total number of surgeries performed by that surgeon in the past year. Both numeric features (years of practice and annual surgical volume) were standardized to normalize their scales. Missing values for these features were imputed using the mean value of the respective column.

Preoperative diagnosis and surgical plan are multilabel categorical variables. The preoperative diagnosis refers to the diagnostic categories assigned to a patient before surgery, and each patient may have 1 or multiple diagnoses. For example, patient A’s diagnosis might be “hydrocele,” whereas patient B’s diagnosis could be “hydrocele, phimosis, and incomplete testicular descent.” Diagnoses were split by commas, and occurrence counts were tallied for each category. To avoid excessive sparsity, only diagnostic categories appearing ≥10 times were retained; others were grouped into a “rare type” category. Multilabel encoding was then applied. For example, “hydrocele” = [1, 0, 0], “phimosis” = [0, 1, 0], “incomplete testicular descent” = [0, 0, 1], and “hydrocele, phimosis, incomplete testicular descent” = [1,1,1].

The “surgical plan” refers to the preoperative plan drafted by the surgeon, and each patient may have 1 or more planned procedures. For example, patient A’s plan could be “bilateral laparoscopic high ligation of the processus vaginalis,” whereas patient B’s plan might be “bilateral laparoscopic high ligation of the processus vaginalis + orchidopexy + circumcision.” Due to differences in writing habits, plans often have inconsistent formatting, with procedure types separated by various delimiters, such as plus signs, commas, or phrases like “simultaneously perform.” Naming conventions for the same procedure were also inconsistent; for instance, “right orchidopexy” and “right-sided orchidopexy” refer to the same surgery. For our prediction target, surgical duration, laterality (“left” vs “right”) was assumed to have no effect for unilateral procedures, so these were combined into “unilateral” categories, whereas “bilateral” procedures were treated separately due to their significant impact on duration. Initially, manual tokenization and grouping were performed based on delimiters (plus signs, commas, etc) and standardization of terms (eg, merging “circumcision” and “circumcision [stapler/ligation/manual suture]”). This yielded 170 categorized procedure types, of which only those occurring ≥10 times were retained. The rest were grouped as “other surgical procedures.” All surgical plans were encoded using the same multilabel approach.

However, due to the high variability in surgical plans, manual tokenization and grouping could not fully resolve inconsistencies, and many equivalent procedures remained uncategorized. To address this, we used an advanced approach using the LLM Qwen3, developed by Alibaba in April 2025. The selection of Qwen over other prominent LLMs (eg, GPT-4 and Llama-3) was driven by its specific alignment with our study’s linguistic and security requirements. Unlike Llama-3, which is primarily optimized for English, Qwen demonstrates superior proficiency in understanding specialized Chinese clinical terminology and semantics relevant to our dataset. Furthermore, in contrast to cloud-based services, such as GPT-4, that necessitate external data transmission, Qwen supports local deployment. This feature allows us to process sensitive patient data entirely within the hospital’s secure intranet, ensuring strict adherence to data privacy regulations while maintaining reasoning and extraction capabilities compared to top-tier proprietary models.

By calling Qwen3’s application programming interface for batch processing, the model summarized all patients’ surgical plans into 23 standardized procedure categories (including “other surgical procedures”). This intelligent consolidation reduced unnecessary feature dimensionality and improved downstream analysis. For example, Qwen3 automatically grouped “penile nevus excision,” “penile cyst excision,” and “penile scar excision” under “penile lesion excision.” Similarly, these standardized categories were then multilabel-encoded for each patient.

Case characteristics and preoperative preparation are unstructured free-text variables describing reasons for admission, degree of organ development, precise lesion locations, and other specifics. Due to similar issues with inconsistent formatting, we again leveraged Qwen3 for structured information extraction. We predefined 4 target features for extraction: prepuce characteristics description, testicular characteristics description, scrotal characteristics description, and penile characteristics description. During extraction, Qwen3 was instructed to output both the structured fields and the corresponding original text segments to guard against hallucinations. Since the extracted text lengths varied, we used the Embedding-3 model (256 dimensions) to produce fixed-length semantic vectors for model input. Embedding-3, developed by ZHIPU AI (Tsinghua University), is a text-embedding model capable of capturing deep semantic relationships and outputting high-quality embeddings. It supports multilingual and long-text inputs, making it well-suited for high-precision semantic representation tasks.

Missing category and text vectors were replaced with null vectors. A detailed description of the surgical feature variables is provided in Table 1.

It is worth noting that while “surgeon name” provides specific identity information, it lacks generalizability, as it is strictly bound to the current roster of physicians and cannot accommodate new surgeons joining the department in the future. To address this limitation, we designed 2 distinct experimental configurations. The first configuration includes “surgeon name” to capture individual-specific variance. The second configuration substitutes “surgeon name” with the quantitative metrics “years of practice” and “annual surgical volume” to construct a more robust and generalizable model capable of predicting surgical duration for new or unobserved surgeons.

**Table 1.** Description of specific surgical feature variables (N=4526).

Patient variables and type	Values/range and statistics
Age (year)	
Numeric	Float (average 5.3, SD 3.7, max 17.0, min 0.0)
Weight (kg)	
Numeric	Float (average 24.6, SD 14.6, max 140, min 5)
Primary surgical procedure	
Categorical	Penile lengthening surgery (2067, 45.6%), laparoscopic high ligation of the processus vaginalis (1158, 25.6%), orchidopexy (937, 20.7%), circumcision (364, 8%)
Lead surgeon	
Categorical	Name: total =31, including “unknown”
Numeric	Years of practice: float (average 16.1, SD 7.1, max 40.6, min 1.5) (y)
Numeric	Annual surgical volume: integer (average 461.2, SD 223.8, max 863, min 2) (cases/y)
Preoperative diagnosis	
Multicategorical	Concealed penis (1839, 40.6%), hydrocele (1202, 26.6%), and cryptorchidism (733, 16.2%).
Surgical plan	
Multicategorical	Penile lengthening (1956, 43.2%), preputioplasty (1649, 36.4%).
Case characteristics and preoperative preparation	
Free text	Prepuce characteristics description: eg, “Phimosis prepuce, can be manually retracted to expose the glans.”
Free text	Testicular characteristics description: eg, “Both testes are located within the scrotum and are well-developed.”
Free text	Scrotal characteristics description: eg, “Scrotum is well-developed.”
Free text	Penile characteristics description: eg, “The penis appears small, with only the distal half of the glans visible on the body surface in the supine position.”
Surgery duration (min)	
Numeric	Float (average 38.6, SD 24.9, max 539, min 4)

### Model Construction

We developed multiple machine-learning methods based on neural networks and traditional models. From the entire dataset of 4526 cases, 15% (n=679 cases) were randomly selected as test data for evaluating the prediction models. The remaining data (referred to as “training data”) were further randomly split into training (n=3168 cases) and validation (n=679 cases) sets at an 85% to 15% ratio. Detailed descriptions of model hyperparameters and validation performance metrics can be found in the Results section. For the finalized model, performance evaluation was conducted only once on the test dataset.

### Pediatric Urology Surgical Duration Prediction Model Based on Multihead MLP

In this study, we trained an MLP model with a multihead structure (multihead MLP) to predict surgical duration in pediatric urology. The core idea is to process different types of input features (text vectors and categorical/numerical vectors) separately through independent subnetworks or branches and then concatenate their outputs and feed them into a shared MLP layer for the final prediction.

**Figure 1.**
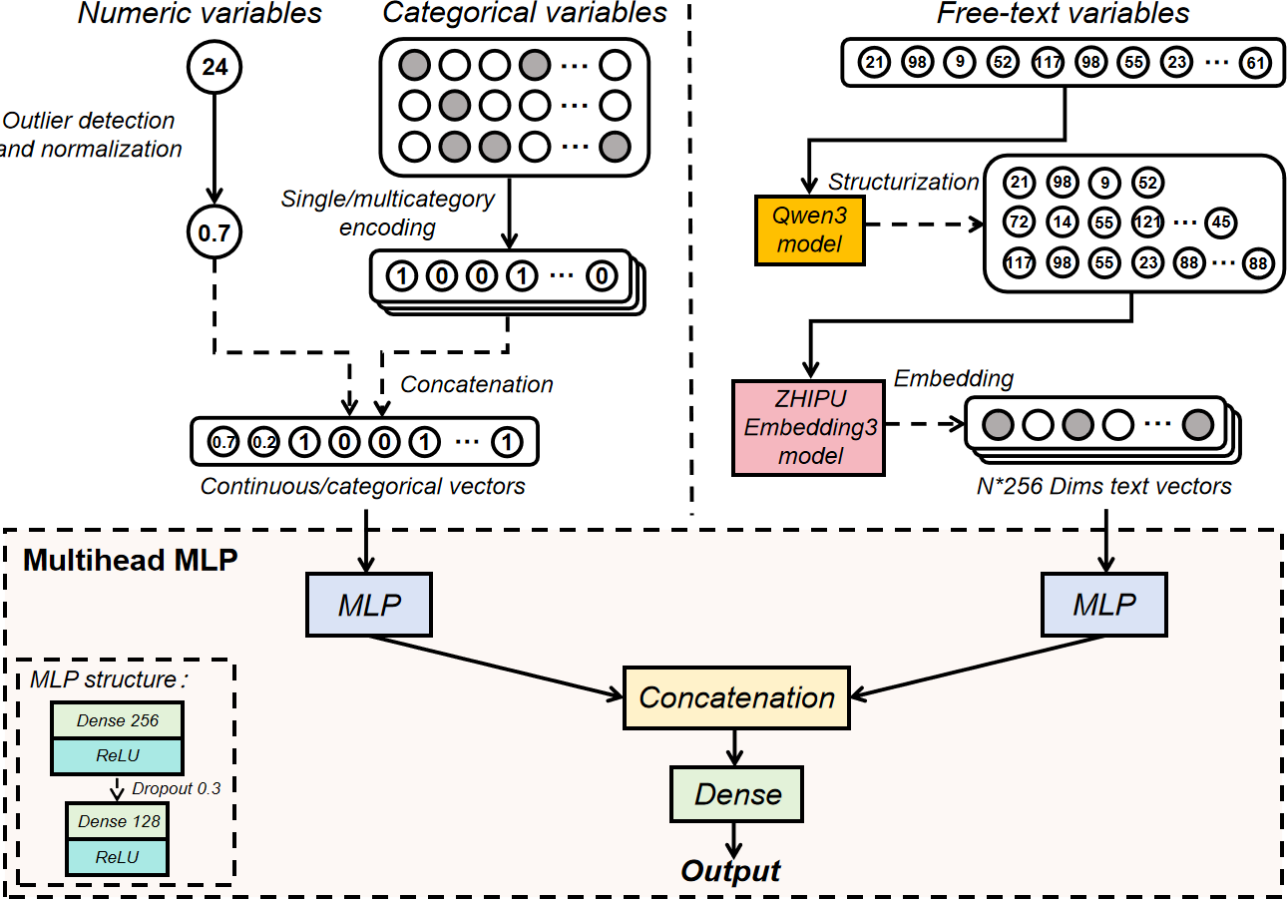
Architecture of the proposed pediatric urology surgical duration prediction model. MLP: multilayer perceptron; ReLU: rectified linear unit.

Traditional single-head MLP concatenates all heterogeneous features into 1 large input vector, which is then passed through a single MLP for prediction. However, due to the severe dimensional imbalance among features in this study (eg, the primary surgery procedure vector is 4-dimensional, while text vectors have dimensions of 4×256), single-head MLP tends to be dominated by features with larger dimensions, drowning out those with smaller dimensions and leading to a tendency for overfitting. To address this, the multihead MLP uses parallel representation learning by establishing independent subnetworks for each data group. This design enables the model to capture the unique patterns and characteristics of each data type separately, thereby reducing interference and ensuring that heterogeneous data are fused within a balanced latent space. To mitigate overfitting, our multihead MLP was trained with early stopping. The architecture of the proposed model is shown in Figure 1.

The multihead MLP architecture comprises 2 distinct subnetworks designed to process heterogeneous input features separately: one dedicated to textual features and the other to categorical and numerical features. Each subnetwork consists of 2 fully connected (dense) layers with 256 and 128 neurons, respectively, each followed by a dropout layer with a rate of 0.3 to mitigate overfitting. Rectified linear unit activation functions are applied throughout. Outputs from both subnetworks are concatenated in a feature fusion layer, which feeds into a subsequent dense layer of 64 neurons accompanied by another dropout layer (rate 0.3). The final output layer uses a single neuron with a linear activation function to produce continuous predictions of surgical duration. The model is optimized using the Adam optimizer with a learning rate of 0.001, trained over 200 epochs, and uses mean squared error as the loss function.

### Model Training and Experimental Settings

This study included a total of 4526 pediatric urology surgical cases with complete recorded surgical times for model development and evaluation. The training dataset (comprising both training and validation sets) contained 3847 cases, including 1730 cases of penile lengthening surgery, 810 cases of orchiopexy, 992 cases of laparoscopic high ligation of the processus vaginalis, and 315 cases of circumcision, accounting for 85% of the total dataset. The test dataset consisted of 679 cases, including 337 penile lengthening surgeries, 127 orchiopexies, 166 laparoscopic high ligations, and 49 circumcisions, representing 15% of the total dataset.

Python 3.8.3 (Python Software Foundation) was used for all feature extraction, algorithm implementation, and performance evaluation. The Alibaba Qwen3 LLM was used for all text analysis and structured field extraction. The ZHIPU AI Embedding-3 large model was used for all text embedding. Google’s TensorFlow 2.4.1 (Google Brain) was used to implement the multihead MLP and other neural networks in comparative experiments. The Scikit-Learn library (scikit-learn community and INRIA) was applied for machine-learning methods, such as decision tree regression (DT) in comparative experiments, with default parameters used for all models.

### Evaluation Metrics

Model performance was assessed using 3 metrics: MAE, RMSE, and the coefficient of determination (R-squared, *R*^2^).

MAE represents the average of the absolute differences between predicted and true values. It directly reflects the average magnitude of prediction errors and is defined by the following formula:

.(1) $$MAE=\frac{1}{n}\sum_{i=1}^{n}|y_{i}-{\hat{y}}_{i}|$$

RMSE is the square root of the average of the squared differences between predicted and true values. It is a commonly used metric to measure the deviation between model predictions and actual values. The formula is as follows:

.(2) $$RMSE=\frac{1}{n}\sum_{i=1}^{n}(y_{i}-{\hat{y}}_{i}{)}^{2}$$

*R*^2^ measures the proportion of variance in the dependent variable (true values) that is explained by the model. It represents the fraction of the total variability in the dependent variable accounted for by the model. An *R*^2^ value of 1 indicates a perfect fit, explaining all variance; *R*^2^=0 means the model explains no more variance than simply predicting the mean; and *R*^2^<0 indicates the model performs worse than predicting the mean. The formula is as follows:

.(3) $$R^{2}=1-\frac{RSS}{TSS}$$

The formulas for the residual sum of squares and the total sum of squares are as follows:

,(4) $$RSS=\sum_{i=1}^{n}(y_{i}-{\hat{y}}_{i}{)}^{2}$$

.(5) $$TSS=\sum_{i=1}^{n}(y_{i}-\bar{y}{)}^{2}$$

where *n* denotes the number of samples, *y*_*i*_ represents the true surgical duration for the *i*th sample, $\bar{y}$ is the mean of all true values, and $\hat{y_{i}}$ is the model’s predicted surgical duration for the *i*th sample.

### Feature Importance Evaluation

Permutation importance is used to assess the significance of individual features within the model. As a model-agnostic method, its core principle is to measure the impact on model performance by randomly permuting the values of a single feature. Initially, predictions are made on the unshuffled test set to establish a baseline performance score. Then, for each feature in turn, its values in the test set are randomly shuffled while keeping other features unchanged, followed by generating new predictions and recalculating the performance score. The difference between the baseline and the shuffled performance indicates the importance of that feature. A significant drop in performance after shuffling a feature implies that the feature is highly influential to the model’s predictive capability.

### Ethical Considerations

From an ethical perspective, this study focuses on the preoperative diagnosis and duration of surgery for patients. The information collection methods adopted by the researchers are designed to ensure that the patients cannot be directly identified, nor can they be indirectly identified through related identifiers. In this study, only the surgery-related content was analyzed, and no personally identifiable information was extracted or associated. Additionally, the identities of the hospital staff involved in the surgery were anonymized and represented only by numerical IDs. This study was performed in line with the principles of the Declaration of Helsinki. All data used in this study were obtained from retrospective surgical cases, and approval was granted by the Academic Ethics Committee of Children’s Hospital Zhejiang University School of Medicine (IRB No. 2023-IRB-0181-P-01; granted on July 20, 2023). We have applied for an informed consent waiver for our study. No compensation was provided to participants, as this was a retrospective study using existing surgical records without direct patient involvement or additional data collection.

## Results

### Accuracy Verification of LLM-Based Extraction

To quantitatively evaluate the fidelity of the structured data generated by Qwen-3 and to assess the prevalence of hallucinations, we conducted a manual validation on a randomly selected subset of 500 cases. This evaluation focused on 4 key features extracted from the unstructured “case characteristics and preoperative preparation” text: prepuce characteristics description, testicular characteristics description, scrotal characteristics description, and penile characteristics description. The validation was performed by a senior medical informatics engineer with over 10 years of experience in clinical data processing. A strict scoring metric (ranging from 0% to 100%) was applied to measure the consistency between the original text and the extracted structured data. Specifically, to rigorously penalize hallucinations, any extraction containing factually incorrect or fabricated information was assigned a score of 0%. Incomplete extractions were scored proportionally based on the extent of the missing information.

**Figure 2.**
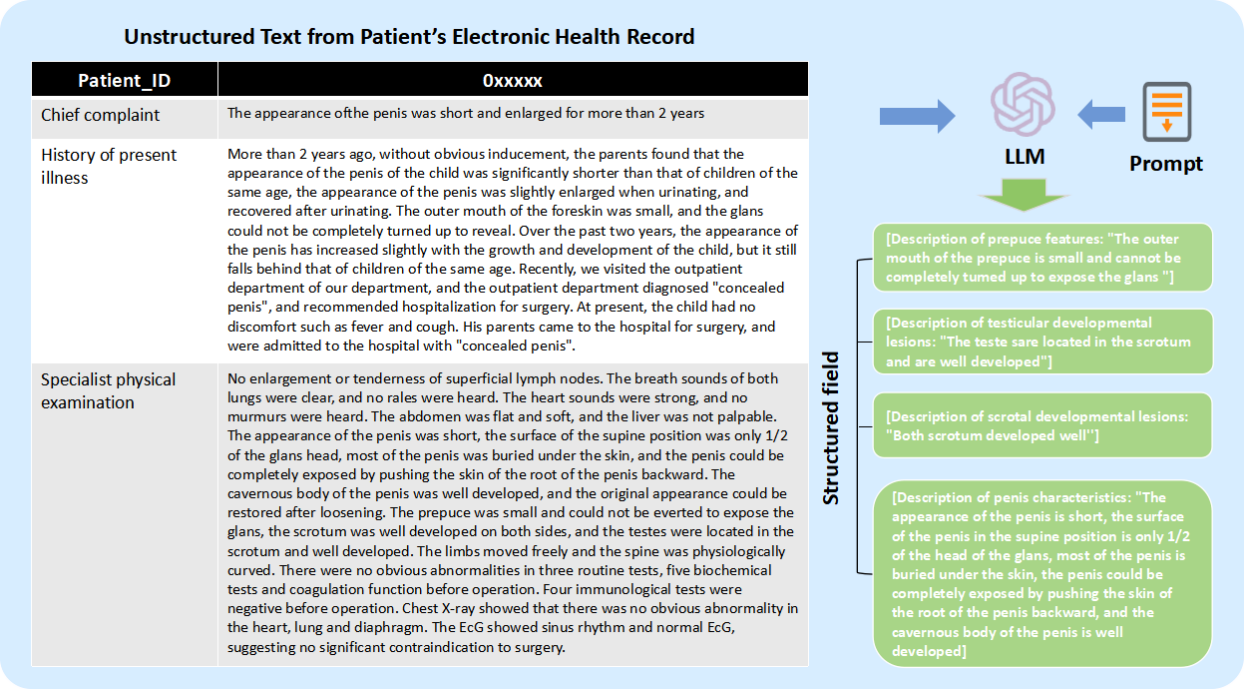
A case of extracting structured fields from patients’ electronic health records text using LLM and prompting. EcG: electrocardiogram; LLM: large language model.

Figure 2 shows a concrete example of extracting structured fields from patient electronic health records using LLMs and prompt text. The evaluation results demonstrated high reliability in the model’s extraction capabilities. The average accuracy scores for the 4 features were 99.6% for prepuce characteristics, 98.94% for testicular characteristics, 93.43% for scrotal characteristics, and 99.18% for penile characteristics. These results confirm that Qwen-3 effectively maintained semantic consistency with the source text while minimizing hallucinations.

### Model Performance Comparison

We compared the proposed method with 6 outstanding surgical duration prediction approaches in the field, including ridge regression (RR) [37], DT [38], support vector regression (SVR) [39], random forest regression (RF) [40], gradient boosting regression tree (GBR) [41], and the machine learning–mixture density network (ML-MDN) model that combines long short-term memory (LSTM) and MLP for surgical duration prediction [23]. All models were trained and tested using the same datasets and feature categories as the proposed model. The RR, DT, SVR, RF, and GBR models used the feature engineering pipeline proposed in this study, whereas the ML-MDN model was implemented using the feature processing strategy described in its original publication. Specifically, the ML-MDN method integrates both structured and unstructured text features using a neural network architecture. In this method, structured variables (categorical and numerical) are encoded or normalized, whereas unstructured text variables (including primary surgical procedure, surgical plan, preoperative diagnoses, and case characteristics) are tokenized and embedded into vectors fed into an LSTM network. The LSTM captures temporal dependencies and contextual information, outputting semantic vectors that are concatenated with structured features and fed into an MDN. The MDN outputs a probability distribution governed by 3 parameters: mean (μ), SD (σ), and mixture coefficients (α). As the other baseline models in this study are deterministic regressors that output single-point estimates, a direct comparison with a probability distribution is infeasible. To ensure a fair and consistent evaluation framework, we used the expected value (mean, μ) of the MDN’s predicted distribution as the representative point prediction. This allows for the standard calculation of MAE, RMSE, and *R*^2^. Additionally, we compared these metrics against a naive baseline (“average”) that simply uses the global mean of the surgical duration from the training set for prediction.

In the first experimental configuration using “surgeon name” as a feature, the proposed method achieved the best performance (MAE=10.74 min, RMSE=14.91, *R*²=0.58). SVR (MAE=11.74 min), RF (MAE=11.62 min), and GBR (MAE=11.68 min) showed comparable, second-best results. RR (MAE=13.06 min) and DT (MAE=15.07 min) demonstrated relatively lower performance. The ML-MDN model’s performance (MAE=16.91 min) was inferior to both our multihead MLP model (MAE=10.74 min) and other traditional machine-learning models. In contrast, the naive “average” baseline, which relied solely on the global mean without utilizing any features or machine-learning algorithms, yielded the poorest performance (MAE=19.06 min). The detailed model evaluation results are shown in Table 2.

**Table 2.** Performance comparison of different surgical duration prediction models and methods on the test dataset utilizing specific surgeon identity “surgeon name”.

Models	MAE^a^	RMSE^b^	*R*²^c^
Average	19.0579	24.8830	0.0000
RR^d^ [37]	13.0554	19.3512	0.2910
DT^e^ [38]	15.0692	21.8759	0.0939
SVR^f^ [39]	11.7400	16.8024	0.4655
RF^g^ [40]	11.6238	16.5873	0.4791
GBR^h^ [41]	11.6821	16.3646	0.4930
ML-MDN^i^ [23]	16.9117	21.4013	0.1328
Multihead MLP^j^	10.7391^k^	14.9134^k^	0.5789^k^

a MAE: mean absolute error.

b RMSE: root mean squared error.

c *R*²: coefficient of determination.

d RR: ridge regression.

e DT: decision tree.

f SVR: support vector regression.

g RF: random forest.

h GBR: gradient boosting regression.

i ML-MDN: machine learning–mixed dense network.

j MLP: multilayer perceptron.

k These values in the last row indicate the best performance among all tested models across each evaluation metric.

In the second configuration, where “surgeon name” was substituted with “years of practice” and “annual surgical volume,” all models exhibited a slight increase in overall error rates. However, the overall performance trend remained consistent: the proposed multihead MLP model still secured the best performance (MAE=11.39 min; RMSE=15.58; *R*²=0.54). Detailed results for this configuration are shown in Table 3.

**Table 3.** Performance comparison of different surgical duration prediction models on the test dataset utilizing generalized surgeon metrics “years of practice” and “annual surgical volume”.

Models	MAE^a^	RMSE^b^	*R*²^c^
Average	19.0579	24.8830	0.0000
RR^d^ [37]	13.6891	20.7138	0.1876
DT^e^ [38]	14.6289	21.9035	0.0916
SVR^f^ [39]	11.8670	16.9053	0.4589
RF^g^ [40]	12.0465	16.8062	0.4652
GBR^h^ [41]	11.6706	16.4278	0.4890
ML-MDN^i^ [23]	17.2747	21.7401	0.1051
Multihead MLP^j^	11.3889^k^	15.5808^k^	0.5404^k^

a MAE: mean absolute error.

b RMSE: root mean squared error.

c *R*²: coefficient of determination.

d RR: ridge regression.

e DT: decision tree.

f SVR: support vector regression.

g RF: random forest.

h GBR: gradient boosting regression.

i ML-MDN: machine learning−mixed dense network.

j MLP: multilayer perceptron.

k These values in the last row indicate the best performance among all tested models across each evaluation metric.

As previously discussed, relying on specific surgeon names limits the model’s applicability to the current roster and fails to accommodate unobserved or newly hired surgeons, thereby reducing generalizability. Therefore, despite the marginal decrease in prediction accuracy, substituting specific identities with quantitative professional metrics (years of practice and annual surgical volume) represents a more robust and scalable solution, ensuring that the model remains effective for future clinical applications. Consequently, all subsequent results and analyses presented in this study utilize “years of practice” and “annual surgical volume” in place of “surgeon name.”

To evaluate the proposed model’s robustness, 5-fold cross-validation was performed. The model demonstrated remarkable stability in point-wise prediction, achieving an average MAE of 11.61 (0.47) minutes, as shown in Table 4. The low SD (0.47 min) indicates that the model’s predictive accuracy is consistent across different data partitions. It is worth noting that while 4 out of 5 folds yielded *R*² scores exceeding 0.50, the second fold showed a performance dip (*R*²=0.28; RMSE=24.95). The discrepancy between the relatively stable MAE (12.05 min) and the spiked RMSE in this fold suggests the presence of extreme outliers (eg, rare complex cases with unexpectedly long durations) in that specific test subset, rather than a systematic failure of the model. Overall, the aggregated results (*R*²=0.47±.11) confirm the model’s generalizability in clinical scenarios.

**Table 4.** Performance metrics of the proposed multihead MLP^a^ model using 5-fold cross-validation.

Models	1-fold	2-fold	3-fold	4-fold	5-fold	Mean (SD)
MAE^b^	11.9191	12.0483	11.8336	10.9581	11.2793	11.6077 (0.4669)
RMSE^c^	16.0396	24.9463	17.6053	16.0877	15.4815	18.0321 (3.9448)
*R*²^d^	0.5291	0.2779	0.5177	0.5396	0.5010	0.4731 (0.1100)

a MLP: multilayer perceptron.

b MAE: mean absolute error.

c RMSE: root mean squared error.

d *R*²: coefficient of determination.

**Figure 3.**
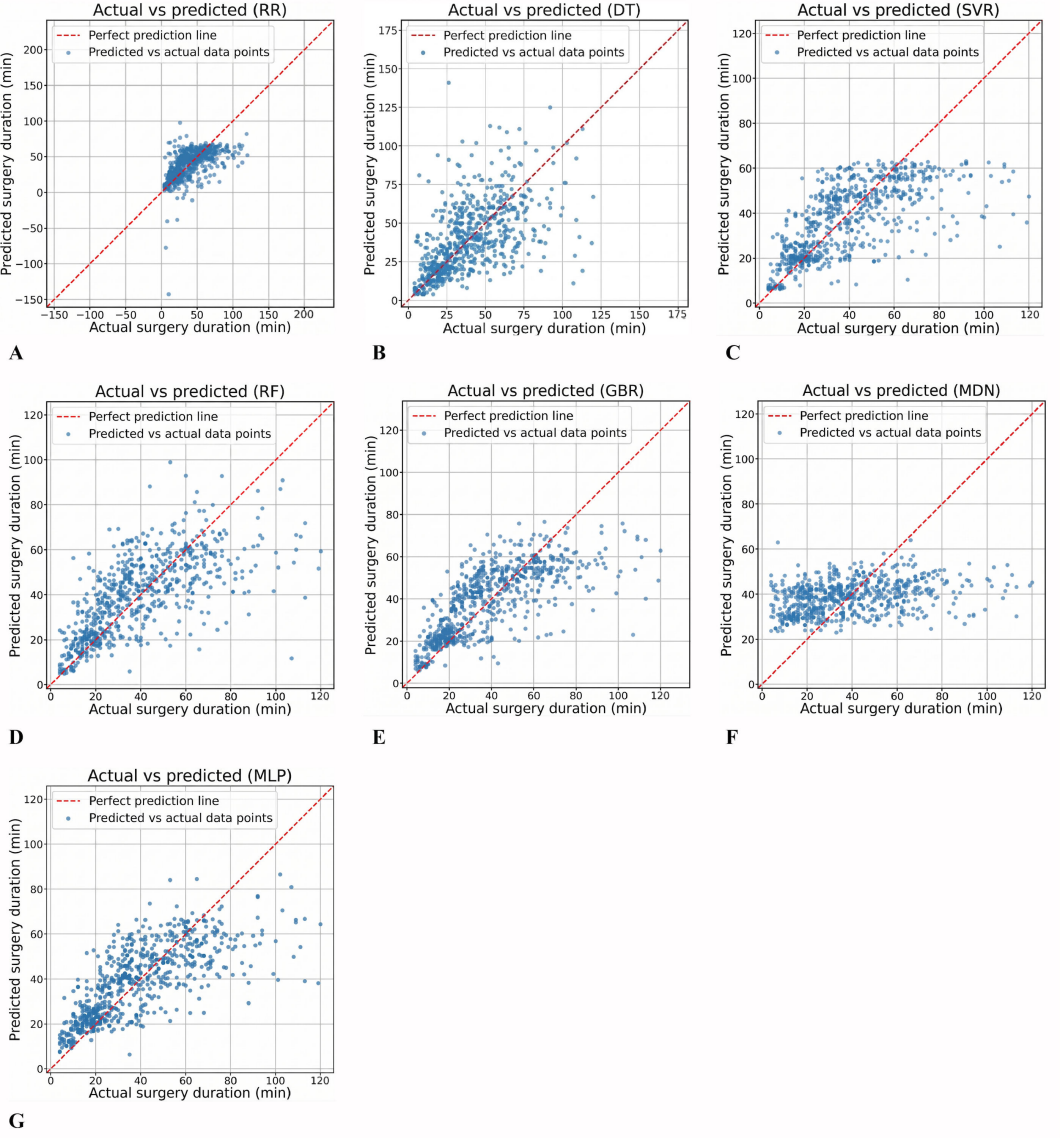
Scatterplots of different models on the test dataset. (A) Ridge regression (RR), (B) decision tree (DT), (C) support vector regression (SVR), (D) random forest (RF), (E) gradient boosting regression (GBR), (F) machine learning–mixture density network (ML-MDN), (G) multihead multilayer perceptron (MLP).

The scatterplot further visualizes the predictive performance of the models, where the red-dashed line represents the ideal prediction—that is, the predicted values equal the true values. The blue scatter points show the distribution of predicted versus actual values in the test set; the closer the points are to the red line, the more accurate the model’s predictions. Scatterplots for all models are shown in Figure 3. It can be observed that the points predicted by RR and DT are highly dispersed (with the largest range on the horizontal axis). Additionally, the ML-MDN model’s predicted points also deviate notably from the red line, consistent with its poorer performance metrics reported earlier. In contrast, the remaining models show less difference, with the multihead MLP exhibiting the most uniform scatter distribution, indicating more stable performance.

### Ablation Study

To demonstrate the effectiveness of the proposed multihead MLP architecture, detailed ablation experiments were conducted on its components. Four different model frameworks were tested separately: single-head MLP with categorical or numerical feature vectors, singlehead MLP with text feature vectors, single-head MLP with concatenated categorical or numerical and text feature vectors, and the multihead MLP. The detailed evaluation results are shown in Table 5. The experimental results indicate that directly concatenating categorical or numerical feature vectors with text feature vectors causes the single-head MLP to be biased toward the higher-dimensional text features, resulting in degraded performance, which is worse than using only the categorical or numerical features. In contrast, the multihead MLP effectively integrates heterogeneous data, leveraging the strengths of features with different dimensions, and achieves the best performance.

**Table 5.** Ablation study on the proposed model architectures and feature combinations.

Different model architectures	MAE^a^	RMSE^b^	*R*²^c^
Single-head MLP *+* categorical/numeric vectors	11.9365	16.4067	0.4903
Single-head MLP^d^ *+* text vectors	12.7383	17.0647	0.4486
Single-head MLP *+* categorical/numeric vectors + text vectors	12.3874	16.9184	0.4581
Multihead MLP + categorical/numeric vectors + text vectors	11.3889	15.5808	0.5404

a MAE: mean absolute error.

b RMSE: root mean squared error.

c *R*²: coefficient of determination.

d MLP: multilayer perceptron.

### Comparison of Performance Using Different Text-Embedding Methods

To further investigate the impact of different text-embedding strategies on model prediction performance, this study designed 2 additional text embedding methods for comparison. The first method uses a traditional tokenization embedding combined with an LSTM network. Specifically, the raw text undergoes preprocessing steps, such as the removal of newline characters and extra whitespace for normalization; the Keras tokenizer layer is then used to tokenize the text and build a vocabulary. The text sequences are padded or truncated to a fixed length and passed through an embedding layer to map tokens into low-dimensional dense vectors. Since the embedding layer only performs initial vector conversion without contextual understanding or complex semantic modeling, the subsequent LSTM layer processes these embedded token vectors to output a semantic vector representing the entire text sequence. The second method directly uses the ZHIPU AI Embedding-3 large model to embed the entire text segment without prior tokenization or Qwen3-based structured preprocessing. This approach treats the text as a whole, aiming to evaluate the large pretrained model’s capacity for text comprehension without explicit preprocessing.

In this experiment, the classification or numerical feature branch was removed, and only the text vectors were used as model input to better assess the influence of different text-embedding strategies on model performance. The results, shown in Table 6, indicate that the method based on the ZHIPU AI Embedding-3 large model outperforms the traditional tokenization + LSTM approach. Furthermore, the Qwen3-based structured preprocessing of the text enhances the embedding model’s performance compared to the direct embedding of unprocessed full text. These findings strongly support the superiority of our proposed LLM-based unstructured text extraction and embedding method in predictive tasks.

**Table 6.** Performance comparison of different text-embedding methods.

Text-embedding methods	MAE^a^	RMSE^b^	*R*²^c^
Tokenizer + embedding layer (Keras) + LSTM^d^	18.7119	22.9938	−0.0010
ZHIPU Embedding-3 model (full text)	12.8774	17.2793	0.4347
ZHIPU Embedding-3 model (structured text)	12.6437^e^	17.0410^e^	0.4502^e^

a MAE: mean absolute error.

b RMSE: root mean squared error.

c *R*²: coefficient of determination.

d LSTM: long short-term memory.

e These values in the last row indicate the best performance among all tested methods across each evaluation metric.

### Feature Importance Evaluation

Permutation importance was used to assess the contribution of each feature within the multihead MLP model. The ranking of feature importance is presented in Multimedia Appendix 1. It is evident that disrupting the feature order of the primary surgical procedure leads to the largest increase in the model’s MAE, indicating that the primary surgical procedure is the most critical feature by a significant margin compared to others. Following this, the surgical plan and preoperative diagnosis rank next in importance, while case characteristics and preoperative preparation, including the unstructured text of the patient’s prepuce description, exhibit the lowest importance.

## Discussion

### Principal Findings

This study demonstrates that an end-to-end surgical duration prediction model integrating heterogeneous multisource data, including structured patient and surgeon characteristics alongside unstructured EMR text, can significantly improve prediction accuracy for pediatric urology procedures. The proposed multihead MLP architecture, which processes categorical, numerical, and textual features through dedicated input branches, effectively addresses the challenge of heterogeneous data fusion and achieves state-of-the-art performance. In comprehensive comparisons against the “average” baseline and 6 established surgical duration prediction methods [23,37-41], our model attains the best results across all metrics (MAE=11.39 min; RMSE=15.58; *R*²=0.54), confirming its advantage in this specialized clinical setting. This improvement is of clear clinical significance. In the dataset used for this study, each operating room typically handles 5 to 8 surgeries per day. Consequently, on a daily cumulative basis, the proposed multihead MLP model reduces the total prediction error by 38.3 to 61.4 minutes compared to the naive “average” baseline. Given that the mean surgical duration is only 38.6 (SD 24.9) minutes, this improvement is substantial, which implies the potential to schedule 1 to 2 additional surgeries, or crucially, to prevent the cancellation of the last patient due to schedule overruns. Furthermore, compared to the traditional ML-MDN model, our approach reduces the daily cumulative error by 29.4 to 47.1 minutes, which is roughly equivalent to the duration of 1 full surgery. Thus, the proposed model directly enhances operating room turnover efficiency and schedule predictability.

### Advantages of Patient-Physician Heterogeneous Feature Fusion

Experimental results indicate that our multisource feature fusion strategy yields significant accuracy improvements over single-source models. Compared to models using only categorical or numerical features or only textual features, we achieve MAE reductions of 4.6% and 10.6%, respectively. The multihead MLP also outperforms the conventional single-head MLP approach by reducing MAE by 8.1%. This enhancement stems from our architecture’s ability to prevent high-dimensional text vectors from dominating the training process at the expense of lower-dimensional features, such as patient age and weight. By providing separate subnetworks for each feature type, our model enables parallel learning across different dimensions, facilitating deeper feature interactions and synergistic enhancement. This design offers valuable methodological insights for addressing similar heterogeneous data integration challenges in medical applications.

### Automated EMR Text Processing Pipeline

Our second major contribution is an automated EMR text processing pipeline leveraging large-scale pretrained models. This approach demonstrates several advantages over traditional tokenization with LSTM networks, including immediate usability, multilingual support, and superior handling of long text inputs. Furthermore, our structured field extraction preprocessing method yields higher-quality embeddings compared to direct processing of raw text segments, confirming the effectiveness of our automated approach in capturing deep semantic relationships within clinical narratives.

### Advantages Over Traditional Methods

In the Results section, we observed that the traditional ML-MDN model (LSTM + MLP) performed significantly worse than our proposed multihead MLP model, falling behind even the simpler RR baseline. This performance discrepancy can be attributed to 3 primary factors: first, the difference in text-encoding mechanisms. ML-MDN relies on LSTMs, which process text sequentially and often struggle with long-range dependencies inherent in verbose medical records. In contrast, Qwen uses a self-attention mechanism, allowing the model to capture global semantic relationships simultaneously and ensuring that critical clinical cues are effectively integrated regardless of their position. This superiority of LLM-based encoding is further corroborated by our ablation study in the Results section. The second factor is the divergence in feature processing strategies. ML-MDN applies a uniform LSTM encoding to all unstructured text fields (including surgical plans and case characteristics). Conversely, our approach adopts a hybrid strategy: we reserve LLM-based encoding exclusively for verbose narrative fields (eg, case characteristics) while using precise multilabel encoding for semistructured concepts, such as surgical plans. The fact that the RR model using our proposed hybrid feature set outperformed ML-MDN serves as strong evidence for the effectiveness of this feature engineering strategy. The third factor is architectural differences. While ML-MDN relies on the simple concatenation of all features, our model uses a multihead input structure. As discussed in the Methods section, this design better handles heterogeneous data, further widening the performance gap.

### Key Predictors and Clinical Insights

Permutation importance analysis identifies the primary surgical procedure as the most influential predictor, aligning with clinical experience that the specific type of surgery is the fundamental determinant of procedural duration. The surgical plan and preoperative diagnosis demonstrated secondary importance, reflecting their roles as key indicators of surgical complexity. Furthermore, the lead surgeon’s years of practice and annual surgical volume also exhibited high predictive value; this finding underscores the necessity of incorporating surgeon-specific metrics into operating room scheduling. Interestingly, textual case descriptions displayed relatively lower importance, likely attributable to the clinical tendency to use standardized language when documenting similar conditions. For instance, descriptions of conditions, such as “small penile appearance” typically adhere to consistent phrasing patterns, limiting their discriminative power. This observation suggests that refining clinical documentation practices could offer opportunities to enhance future predictive modeling.

### Limitations and Further Research Directions

A primary limitation of this research stems from its reliance on data obtained from a single tertiary pediatric hospital. While the dataset exhibits strong internal consistency and an adequate sample size, the single-center provenance may nonetheless constrain the model’s generalizability across other health care settings, particularly where disparities exist in clinical workflows, surgical practices, or electronic health record documentation standards. Such limitations are frequently encountered in medical artificial intelligence applications. Future efforts could involve validating model robustness through multicenter collaborations and exploring approaches, such as domain adaptation or lightweight transfer learning. For instance, fine-tuning models with limited annotated data from target institutions improve applicability across diverse clinical environments.

Moreover, the current predictive model relies exclusively on preoperative data, whereas actual operative duration is frequently influenced by intraoperative factors that cannot be anticipated. For example, the incidental identification of contralateral cryptorchidism may necessitate a shift from a unilateral to a bilateral procedure, while unforeseen events, such as anatomical anomalies or intraoperative bleeding, can extend surgical time. Such dynamic intraoperative developments fall outside the scope of static preoperative variables and may introduce deviations in predictions. To this end, we plan to build a 2-stage prediction framework in future work: the first stage generates baseline estimates using preoperative data, and the second stage dynamically corrects these estimates by integrating real-time information, such as laparoscopic video streams, anesthesia records, and instrument usage logs, after the operation begins. This approach has the potential to maintain the efficiency of preoperative planning while improving the adaptability and precision of resource allocation during surgery.

### Conclusion

This study tackles the clinical challenge of surgical duration prediction in pediatric urology by introducing a specialized predictive model that leverages an innovative multimodal data fusion approach. Our findings demonstrate that combining LLM-based text processing with a multihead MLP architecture successfully integrates structured clinical data with unstructured medical narratives, leading to a significant improvement in prediction accuracy. The model outperforms existing methods across key performance metrics, including MAE and RMSE, confirming the value of heterogeneous feature collaboration in predictive modeling. Feature importance analysis identifies critical predictors, such as the primary surgical procedure, surgical plan, and preoperative diagnosis, offering evidence-based insights for clinical resource allocation. Additionally, sensitivity analysis underscores the importance of pediatric-specific clinical characteristics, including developmental stage variations and urinary tract malformation severity, reinforcing the need for specialty-tailored modeling approaches. These results provide reliable decision-support for operating room scheduling and establish a methodological framework for surgical duration prediction in other specialized fields.

While the study yields promising results, certain limitations must be acknowledged, particularly the reliance on single-institution data. Future research should prioritize multicenter data collection to enhance model generalizability, investigate dynamic prediction methods that incorporate real-time intraoperative data, and develop more interpretable tools for analyzing feature interactions. Such refinements would further strengthen the model’s applicability in complex clinical settings. Overall, this work presents a practical and effective technological solution for intelligent surgical management in pediatric subspecialties, with meaningful implications for optimizing operating room efficiency and health care resource use.

## Supplementary material

10.2196/82329 Multimedia Appendix 1 Feature importance ranking of different variables in the proposed surgical duration prediction model.

## Abbreviations

DT: decision tree regression
EMR: electronic medical record
GBR: gradient boosting regression tree
LLM: large language model
LSTM: long short-term memory
MAE: mean absolute error
ML-MDN: machine learning–mixture density network
MLP: multilayer perceptron
RF: random forest regression
RMSE: root mean square error
RR: ridge regression
SVR: support vector regression
