# Supplementary figures and images for: Predicting Pediatric Urological Surgery Duration Through Multimodal Patient-Physician Feature Fusion: Deep Learning Framework Incorporating Clinical Text Embedding
Source: JMIR Med Inform. 2026 Apr 28;14:e82329. doi: 10.2196/82329 (PMC13123755; doi:10.2196/82329)

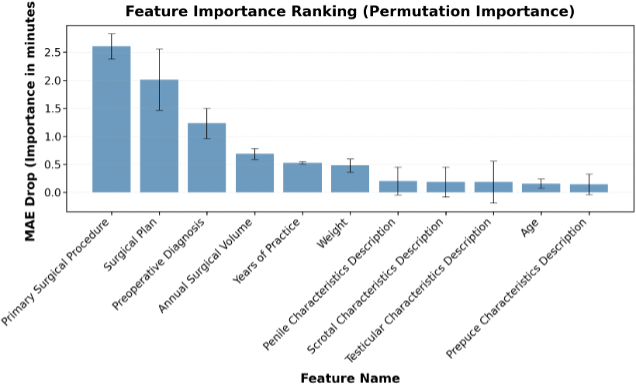

Supplement: Multimedia Appendix 1 [file medinform-v14-e82329-s001.png]
